# Soluble CD52 mediates immune suppression by human seminal fluid

**DOI:** 10.3389/fimmu.2024.1497889

**Published:** 2024-12-16

**Authors:** Leonard C. Harrison, Natalie L. Stone, Esther Bandala-Sanchez, Nicholas D. Huntington, Robert I. McLachlan, Jai Rautela, Moira K. O’Bryan

**Affiliations:** ^1^ Population Heath and Immunity Division, Walter and Eliza Hall Institute of Medical Research, Parkville, VIC, Australia; ^2^ The University of Melbourne, Department of Medical Biology, Parkville, VIC, Australia; ^3^ Hudson Institute of Medical Research, Monash University, Melbourne, VIC, Australia; ^4^ School of Biosciences and Bio21 Molecular Science and Biotechnology Institute, Faculty of Science, The University of Melbourne, Melbourne, VIC, Australia

**Keywords:** seminal fluid, CD52, HMGB1, Siglec-7, T cell, NK cell

## Abstract

Seminal fluid provides for the carriage and nutrition of sperm, but also modulates immunity to prevent allo-rejection of sperm by the female. Immune suppression by seminal fluid has been associated with extracellular vesicles, originally termed prostasomes, which contain CD52, a glycosylated glycophosphoinositol-anchored peptide released from testicular epithelial cells. Previously, we reported that human T cell-derived CD52, bound to the danger-associated molecular pattern protein, high mobility group box 1 (HMGB1), suppresses T cell function via the inhibitory sialic acid-binding immunoglobulin-like lectin-10 (Siglec-10) receptor. Here we show that human seminal fluid contains high concentrations of CD52 complexed with HMGB1, which mediates T cell suppression indirectly via Siglec-7 on antigen-presenting cells. Proliferation of natural killer (NK) cells, which express Siglec-7 and play a key role in the immune defence of the uterus, was directly suppressed by seminal fluid CD52. These findings elucidate a critical function of seminal fluid to suppress cellular immunity and facilitate reproduction.

## Introduction

Since the first reports of suppression of lymphocyte activation (1, 2), immune modulation by seminal fluid has been ascribed to prostaglandins, spermine, complement inhibitors, soluble Fc receptors and transforming growth factor (TGF)-β, and associated with membrane-bound extracellular vesicles (EVs) originally termed prostasomes (reviewed in 3, 4). Initially, inhibition of lymphocyte activation and NK cell function was attributed to prostaglandins of the E series (PGEs) present in high concentration in seminal fluid (5–7). Seminal fluid was then shown to contain TGF β (8), which elicited protective immune responses in the female and, in concert with PGEs, deviated T-cell differentiation towards Th2 (IL-4-secreting) or Th3 (IL-10-secreting) T cells (9). In addition, inhibition of lymphocyte function was also shown to be associated with prostasomes, but the molecular mediator was not identified (10). Prostasomes contain several glycophosphatidylinositol (GPI)-linked membrane proteins, including CD52 and the complement inhibitor CD59 (11).

CD52 is a small glycoprotein attached by a GPI anchor to the surface of lymphoid cells, originally identified as the target of the lymphocyte-depleting rat monoclonal antibody Campath (12, 13). CD52 is also found in the male reproductive tract, where it is released by epithelial cells of the epididymis into the seminal fluid and becomes incorporated into the sperm cell membrane (14) and prostasomes (11). Prostasomes are a heterogenous population of EVs, reflecting different cells of origin. Those derived from the epididymal epithelium, and containing CD52 (11), have been referred to as epididymosomes (15). The structure of the N-linked sialoglycan of CD52 on sperm is similar, but not identical, to that on T cells (16–18). CD52 on sperm was shown to be the target of sperm-immobilizing antibodies in the serum of a minority of infertile women (19).

We reported that soluble CD52 is released from activated human CD4^+^ T cells and suppresses T cells by binding to the inhibitory receptor, sialic acid-binding immunoglobulin-type lectin (Siglec)-10 (20), expression of which is increased upon T cell activation (21). Furthermore, we found that to bind Siglec-10 soluble CD52 had to first complex with the pro-inflammatory Box B domain of high-mobility group box 1 (HMGB1) (22), a damage-associated molecular pattern (DAMP) protein present in serum (23). Of interest, an early study (6) found that the effect of human seminal fluid to suppress T cell activation required the presence of serum in the culture medium. Soluble lymphoid CD52 also suppressed NF-kB-mediated signalling by innate immune cells (monocytes, macrophages, dendritic cells), which required HMGB1 but did not appear to be mediated by Siglec-10 (24). In the present study, we show that soluble CD52 bound to HMGB1 largely accounts for the capacity of human seminal fluid to suppress the activation and proliferation of T and NK cells.

## Materials and methods

### Samples

Coded, anonymous donor semen samples were provided in several batches by Monash IVF, Melbourne, under approval by Monash Health Human Research Ethics Committee (#15172 M). Samples chosen at random were centrifuged at 10,000 x g for 10 min to pellet sperm cells. Seminal fluid supernatants were inspected microscopically to ensure the absence of cells, and stored at -80°C. Heparinised blood samples were obtained with informed consent from healthy adult donors in the Walter and Eliza Hall Institute (WEHI) Volunteer Blood Donor Registry and studies were approved by the WEHI Human Research Ethics Committee.

### Antibodies and other reagents

Mouse monoclonal antibody (mAb) to human CD52 (clone CF1D12) and its IgG3 isotype control (against a Plasmodium falciparum antigen), and Protein G-Sepharose, were supplied by the WEHI Monoclonal Antibody Facility; rat mAb to CD3 (clone OKT3) and mouse mAbs to CD28 (clone 28.2), human CD4 (clone RPA-T4) and human CD52 (IgM, cat# 338202) were from Biolegend (San Diego, CA); mouse mAbs to human Siglec-7 (clone QA79), human Siglec-9 (clone K8) and human Siglec-10 (clone 5G6), unconjugated and phycoerythrin (PE)-conjugated, and to CD56 (clone MEM-188) fluorescein isothiocyanate-conjugated, were from Thermo-Fisher (Scoresby, VIC, Australia); goat affinity-purified polyclonal antibodies to human Siglec-7 and Siglec-10 were from R&D Systems (Minneapolis, MN). Alemtuzumab, the humanized form of Campath-1 rat mAb to human CD52, which binds to the junction of the CD52 peptide and its GPI anchor in lymphoid CD52 (12), was from Bayer Healthcare (Pymble, Australia); rabbit polyclonal HRP-labelled antibody to HMGB1 (ab128129) was from Abcam (Cambridge, UK); mouse mAb to human IFN-γ (clone 1-D1K) was from Mabtech (Nacka Strand, Sweden); Mini-Leak Medium matrix, divinyl sulfone-activated agarose, was from Kem-En-Tec (Copenhagen, Denmark). Anti-CD3/CD28 Dynabeads were from ThermoFisher. Other reagents included: enhanced chemiluminescence (ECL) kit (GE Healthcare, Rydalmere, NSW, Australia), anti-human CD3-FITC, anti-human CD4-biotin, anti-FITC, anti-biotin microbeads and LS columns (Miltenyi Biotec, North Ryde, NSW, Australia), FlowCheck beads (Beckman Coulter, Gladesville, NSW, Australia), carboxyfluorescein diacetate succinimidyl ester (CFSE), bovine serum albumin (A7906) (BSA), 3,3’,5,5’-tetramethylbenzidine (TMB) and horse radish peroxidase (HRP) substrate solution (T2885) (Sigma-Aldrich, Sydney, NSW, Australia), ^3^H-thymidine (ICN, Sydney, NSW, Australia), and tetanus toxoid (tetanus) (generously provided by CSL, Parkville, VIC, Australia). Recombinant CD52-Fc fusion protein was produced in Expi293 cells and purified as previously described (20).

### CD52 ELISA

Mouse IgM anti-human CD52 capture antibody at 5 μg/ml in phosphate-buffered saline (PBS) was added (50 μl) to wells of a Nunc Maxisorp plate (ThermoFisher) and incubated overnight at 4°C. Wells were washed x3 with PBS-0.05% Tween-20 (PBST), then x3 with PBS. Blocking solution, 5% BSA in PBS, was added (150 μl/well) for 1 h at room temperature (RT), and wells washed again. Dilutions of seminal fluid (in 50 μl) were added in triplicate wells and incubated at RT for 3 h; blanks were blocking solution only. Wells were washed again before addition of horse radish peroxidase (HRP)-labelled alemtuzumab anti-CD52 antibody (2.8 μg/ml) in 5% BSA-PBS (100 μl/well). After 1.5 h at RT, wells were washed, before addition of chromogenic TMB substrate solution (100 μl/well). Colour development was terminated by addition of 0.5M H_2_SO_4_ (50 μl/well), and absorbance read at 450nm in a Thermo Labsystems Multiskan Ascent spectrophotometer.

### Immunodepletion of CD52

Protein G-Sepharose (100 mg) in 35 μl PBS was mixed with 1 mg anti-CD52 Ab (alemtuzumab) or 1 mg control IgG each in 35 μl PBS on a rotator for 2 h at RT. After centrifugation, the pellets were washed x3 in PBS and resuspended in 200 μl PBS. 20 μl seminal fluid diluted 1:2 in PBS was added to each, before rotation for 2 h at 4°C. After centrifugation, the supernatant was saved and the pellet boiled in SDS sample buffer and retained for immunoblotting.

Alternatively, alemtuzumab (3 mg) or control human immunoglobulin was covalently coupled to Mini-Leak agarose beads (1ml) overnight at RT, blocked and washed, according to the manufacturer’s instructions. The beads were incubated with 160 μl seminal fluid + 640 μl PBS by gentle rotation overnight at 4°C. After centrifugation, the supernatant was collected, analysed by Western blotting and tested in cell assays.

### Immunoblotting

Proteins were fractionated in a 4-20% NuPAGE gel (ThermoFisher) and blotted onto PVDF membranes. Membranes were washed in Tris-buffered saline with 0.1% Tween 20 detergent (TBST) then blocked in TBST-5% skim milk powder for 1 h at RT, before incubation for 1 h at RT with horseradish peroxidase (HRP)-labelled alemtuzumab (1.5 mg/ml) diluted in blocking buffer. After washing in TBST, proteins were detected by ECL chemiluminescence.

### Ultracentrifugation

Semen was initially centrifuged at 10,000 x g for 10 min to remove sperm cells. The seminal fluid supernatant was then ultracentrifuged for 1 h at 100,000 x g to deplete extracellular vesicular prostasomes (10, 11). The pellet containing prostasomes was reconstituted to the original volume and together with the soluble supernatant tested for suppression of tetanus-stimulated IFN-γ production.

### Cells

Peripheral blood mononuclear cells (PBMCs) were isolated from heparinised blood on Ficoll/Hypaque (Amersham Pharmacia, Uppsala), washed in phosphate buffered saline (PBS) and resuspended in Iscove’s modified Dulbecco’s medium (Gibco, Melbourne, Australia) containing 5% pooled, heat-inactivated human AB serum, 100 mM non-essential amino acids, 2 mM glutamine and 5 x 10^-5^ M 2-mercaptoethanol (IP5 medium). The Raji (human Burkitt B-cell lymphoma) and Jurkat (CD4^+^ human T-cell leukaemia) cell lines were obtained from the WEHI Monoclonal Antibody Facility. CD4^+^ T cells were purified from PBMCs by negative immunomagnetic selection with a Dynabeads™ Untouched Human CD4 T Cell Kit (ThermoFisher). Following purification, flow cytometry demonstrated that CD4^+^ T cells were >96% pure. Untouched NK cells were purified from freshly prepared PBMCs by immunomagnetic negative selection (Miltenyi Biotec) and suspended in NK MACS medium (Miltenyi Biotec) containing 5% pooled, heat-inactivated human AB serum.

### Dye dilution T-cell division assay

PBMCs from healthy donors were labelled with the cell division-tracking dye CFSE (0.1 μM) and cultured in IP5 medium at 2x10^5^ in 200 μl/well of round-bottom 96-well plates for 7 days, in triplicate with different dilutions of seminal fluid and other agents as shown. Viable (propidium iodide negative) CD4^+^ T cells that had divided (CFSE^dim^) were analysed in a FACSAria (BD Biosciences) and the cell division index (CDI) calculated, based on the number of CD4^+^ cells that had divided per 20,000 undivided CD4^+^ cells (25).

### Enzyme-linked immunospot assay

PBMCs (2x10^5^/well) or purified CD4^+^ T cells 1x10^4^/well) were incubated in 200 μl IP5 medium in replicates of three in 96-well ELISpot plates (MultiScreen HTS, Millipore, Bayswater, VIC, Australia) for 18-24 h at 37°C in 5% CO_2_ air. Wells had been conditioned by washing with 35% ethanol before being coated with anti-human IFN-γ mAb (10 μg/ml) in PBS overnight at 4°C. PBMCs were incubated with tetanus (10 LFU/ml) and PBMCs or purified CD4^+^ T cells with anti-CD3/28 Dynabeads (1 bead/cell) +/- seminal fluid. After 24 h, cells were lysed with water and discarded. Wells were washed with PBS between sequential incubations with biotinylated anti-human IFN-γ (1μg/ml), streptavidin-alkaline phosphatase (Mabtech) and 5-bromo-4-chloro-3-indolyl-phosphate/nitro blue tetrazolium substrate solution (Mabtech). The colour reaction was stopped by addition of water and IFN-γ spots counted with an AID ELISpot Reader (Autoimmun Diagnostika Gmbh, Strassberg, Germany).

### ^3^H-thymidine uptake assay

Jurkat leukaemia CD4^+^ T cells or Raji human B-cell lymphoma cells, grown to ~4x10^6^ cells/ml in RPMI/10% FCS medium, were incubated with seminal fluid (1/40 dilution) in a total volume of 200 μl, at 37°C in 5% CO_2_-air, as described in figure legends. ^3^H thymidine (37 kBq) was added to wells during the last 16h of incubation; the cells were then collected on glass fibre filters, washed, dried and counted in scintillant in a beta-counter to measure proliferation.

### Statistics

Except for measurement of T-cell division by CFSE dye dilution, experiments were performed in replicates of 3 or 6 and results expressed as mean ± standard deviation (SD). Significance between groups was determined by unpaired or paired t tests (2-tail) using GraphPad Prism (GraphPad Software Inc., San Diego, CA).

## Results

### Seminal fluid contains high concentrations of CD52

**Figure 1:**
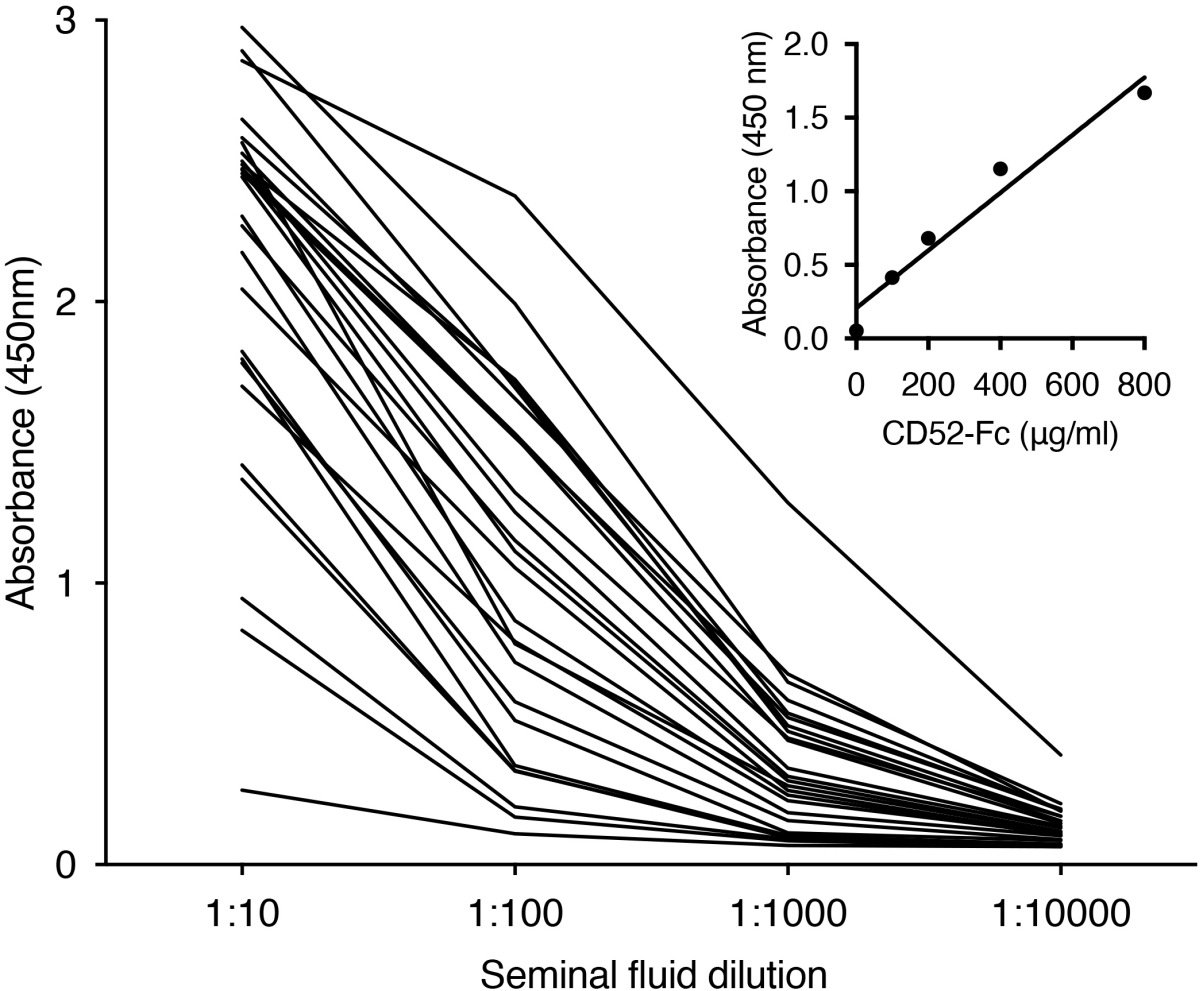
Seminal fluid contains high concentrations of CD52. Individual seminal fluid samples (n=25) were diluted as shown and assayed for CD52 by ELISA. Inset: titration of recombinant human CD52-Fc.

CD52 was previously identified in seminal fluid by immunoblotting and flow cytometry (26) but not directly quantified. By ELISA, we detected CD52 in seminal fluid out to 1:10,000 dilution (**Figure 1**). Its concentration varied widely but appeared to be remarkably high overall compared to recombinant human CD52-Fc dimer as a ‘standard’ (**Figure 1**, inset). Absolute quantitation was not possible because the glycosylation and immunoreactivity of native compared to recombinant CD52 is unknown; furthermore, the molecular mass of CD52 cannot be determined accurately because it is predominantly glycan, and its small peptide does not react with protein staining reagents. Nevertheless, the mean absorbance ± SD at 1:100 dilution (in the linear section of the dilution curves) is 1.053 ± 0.626. Read off the CD52-Fc ‘standard curve’, this equates to an equivalent CD52-Fc concentration of 420 μg/ml, or 42 mg/ml after correcting for dilution. However, CD52 comprises only 3% of the CD52-Fc construct and when this is taken into account the CD52-Fc equivalent concentration = 1,260 μg/ml.

### Seminal fluid CD52 is bound to HMGB1

The immune suppressive activity of recombinant CD52-Fc requires its binding to HMGB1 (22). We surmised therefore that if CD52 contributed to the immune suppressive activity of seminal fluid then HMGB1 may be present in seminal fluid. Immunoprecipitation with anti-CD52 antibody, alemtuzumab, followed by blotting with anti-HMGB1 antibody demonstrated that HMGB1 is present and bound to CD52 in seminal fluid (**Supplementary Figure 1**). Immunoreactive HMGB1 was not detected after depleting CD52 from seminal fluid, indicating that CD52 is in relative excess and able to bind all available HMGB1.

### Seminal fluid suppresses T-cell proliferation and function

**Figure 2:**
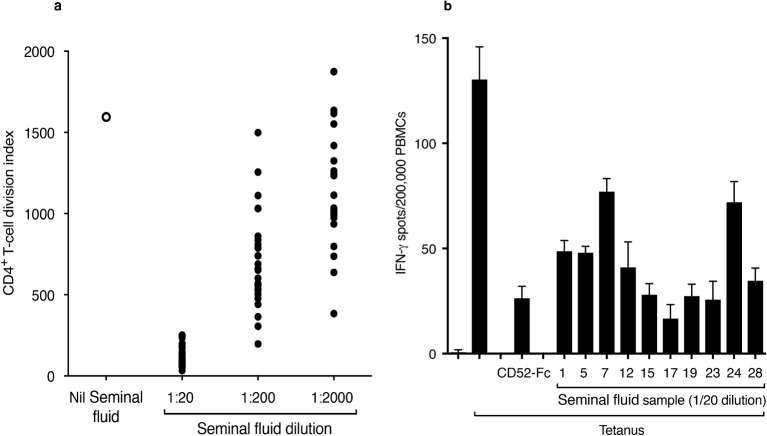
Seminal fluid suppresses T-cell proliferation and function. **(A)** Proliferation of CD4^+^ T cells in response to tetanus in the absence or presence of 26 individual, serially diluted seminal fluid samples. PBMCs were labelled with CFSE dye and incubated for 7 days in the absence or presence of tetanus toxoid (10 LFU/ml) and seminal fluid samples. After staining for CD4, divided (CFSE^dim^) CD4^+^ cells were expressed as the cell division index. **(B)** IFN-γ ELISpots in PBMCs in response to tetanus in the absence and presence of individual seminal fluid samples. PBMCs were incubated in triplicate in an ELISpot plate coated with anti-IFN-γ antibody, for 18 h at 37°C in 5% CO_2_ air, in the presence of tetanus (10 LFU/ml) and seminal fluid samples. CD52-Fc (10 μg/ml) was included as a positive control. Data are mean ± SD.

Multiple individual seminal fluid samples diluted out to 1:200 or more suppressed proliferation of CD4^+^ T cells in response to tetanus, determined by dilution of CFSE dye labelled PBMCs (**Figure 2A**) and suppression of IFN-γ production by PBMCs in response to tetanus, determined by ELISpot assay (**Figure 2B**).

### CD52 mediates T-cell suppression by seminal fluid

**Figure 3:**
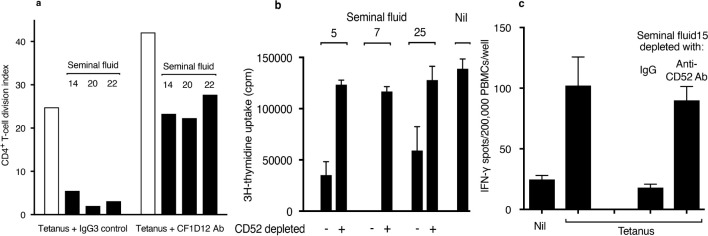
Blocking or depletion of CD52 prevents T-cell suppression by seminal fluid. **(A)** CD4^+^ T-cell proliferation in PBMCs measured by CFSE dye dilution (see **Figure 2A**) in response to tetanus in the presence of seminal fluid and either IgG3 isotype control or CF1D12 (10 μg/ml) anti-CD52 glycan antibody. **(B)** Proliferation of Jurkat T cells incubated in triplicate for 48 h in the presence of seminal fluid (final dilution 1:40) ‘depleted’ by either agarose-bound human control IgG or humanized anti-CD52 antibody, alemtuzumab. ^3^H-thymidine was added for the last 16 h of incubation, and the cells then washed and analysed by scintillation counting. Data are mean ± SD. **(C)** IFN-γ ELISpots in PBMCs in response to tetanus (see Methods) in the presence of seminal fluid (final dilution 1:40) ‘depleted’ by either agarose-bound human control IgG or alemtuzumab anti-CD52 antibody. Data are mean ± SD. Individual seminal fluid samples are numbered.

The contribution of CD52 to T-cell suppression by seminal fluid was examined by antibody blocking and immunodepletion. CF1D12 is a mouse monoclonal antibody directed to the bioactive glycan moiety of lymphoid CD52 (13). In the presence of CF1D12, suppression by seminal fluid of CD4^+^ T-cell proliferation in PBMCs in response to tetanus was abolished (**Figure 3A**). In the absence of seminal fluid, CF1D12 alone increased CD4^+^ T-cell proliferation in response to tetanus, which we attribute to blocking by CF1D12 of soluble CD52 released upon T-cell activation (20). The humanized anti-CD52 antibody, alemtuzumab, coupled covalently to Mini-Leak agarose beads depleted almost all CD52 from seminal fluid, as determined by immunoblotting (**Supplementary Figure 2**). Suppression by seminal fluid of proliferation of Jurkat human CD4^+^ T-cell leukaemia cells (**Figure 3B**) or the IFN-γ response of PBMCs to tetanus (**Figure 3C**) was not observed after depletion of CD52.

### Immune suppression is mainly associated with the soluble seminal fluid compartment

Previously, suppression of lymphocyte proliferation by seminal fluid was shown to be associated with prostasomes (10), which were later found to contain CD52 (11). Initially, to clarify seminal fluid we centrifuged samples at 10,000 x g for 10 min to remove sperm cells, but this would not have depleted prostasomes (10). To determine the contribution of prostasomes to immune suppression attributed to CD52, compared to the soluble compartment of seminal fluid, we subjected seminal fluid samples to ultracentrifugation, which depletes prostasomes (10, 11). The pellet containing prostasomes was reconstituted to the original volume and together with the soluble supernatant tested for suppression of tetanus-stimulated IFN-γ production. Compared to the original non-centrifuged seminal fluid, suppressive activity was present predominantly in the supernatant (**Supplementary Figure 3**). This result is like that of Rooney et al. (11), who showed that while some GPI-linked glycoproteins were associated with prostasomes in seminal fluid the majority of CD52 was in a soluble, prostasome-free form.

### Siglec-7 mediates immune suppression by seminal fluid CD52

Previously, we reported that lymphoid-derived soluble CD52 complexed with HMGB1 suppressed T-cell function by binding the inhibitory Siglec-10 receptor on T cells (20, 22). The N-linked sialoglycan of seminal fluid CD52 is structurally distinct from that of lymphoid CD52 (15–17) and therefore we sought to identify the potential Siglec target for seminal fluid CD52. Initially, we screened several cell lines for expression of multiple Siglecs. While primary human B cells express Siglecs-2, -5, -6 and -10 (27) we found that the Raji human B-cell lymphoma line also expressed Siglecs 7 and 9 (**Supplementary Figure 4**).

**Figure 4:**
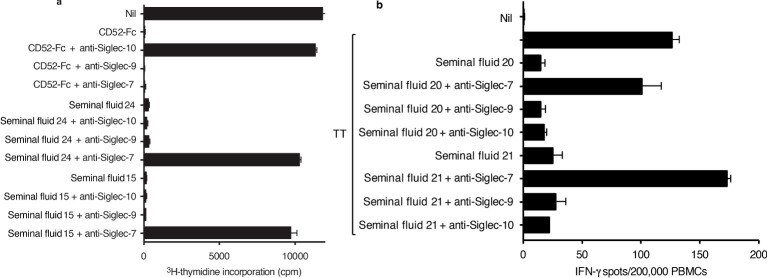
Siglec-7 mediates immune suppression by seminal fluid. **(A)** Raji human B-cell lymphoma cells (1x10^5^ per flat bottom well) were incubated in triplicate with CD52-Fc (10 μg/ml) or seminal fluid samples (1/40 dilution) +/- anti-Siglec 10, -9 or -7 antibodies (10 μg/ml) for 9 days. ^3^H thymidine was added to wells during the last 16 hours of incubation; the cells were then collected on glass fibre filters, washed, dried and counted in scintillant in a beta-counter. **(B)** PBMCs were incubated in triplicate with tetanus (10 LFU/ml) and seminal fluid (1/40 dilution) +/- anti-Siglec-7, -9 or -10 antibody (10 μg/ml) in ELISpot plates pre-coated with IFN-γ antibody, for 24 h at 37°C in 5% CO_2_ air, before development of IFN-γ spots. Data are mean ± SD.

In the presence of either CD52-Fc or seminal fluid, proliferation of Raji cells was almost totally suppressed (**Figure 4A**). Suppression by CD52-Fc was prevented by antibody to Siglec-10, whereas suppression by seminal fluid was prevented by antibody to Siglec-7, but not by antibody to Siglec-9, which is 84% identical to Siglec-7 (28). Although the Raji cell line identified Siglec-7 as mediating suppression by seminal fluid CD52, Siglec-7 RNA or protein expression is insignificant in primary human B cells (27; proteinatlas.org). Suppression by seminal fluid of the IFN- γ response to tetanus in PBMCs was also prevented by anti-Siglec-7, but not by anti-Siglec-9 or -10antibody (**Figure 4B**). This difference in specificity of Siglec receptors is consistent with seminal fluid and lymphoid CD52 being structurally distinct.

**Figure 5:**
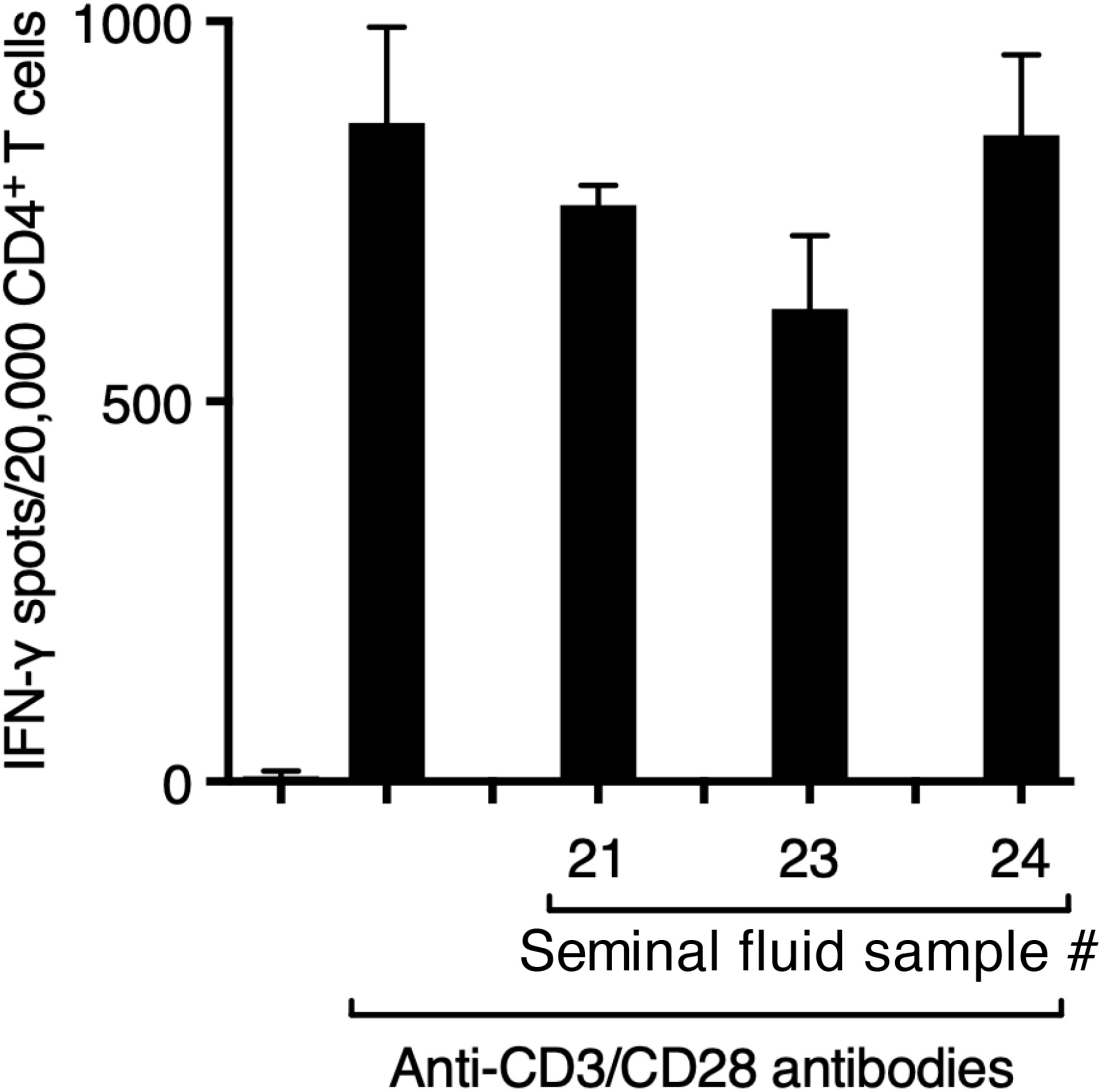
Seminal fluid does not suppress the function of purified CD4^+^ T cells. CD4^+^ T cells (5x10^3/^well), >96% pure by negative immunomagnetic selection, were incubated with anti-CD3/CD28 Dynabeads (1 bead/cell) in the presence of seminal fluid (final dilution 1:20) in ELISpot plate wells pre-coated with antibody to IFN-γ for 24 h, before development of IFN-γ spots.

**Figure 6:**
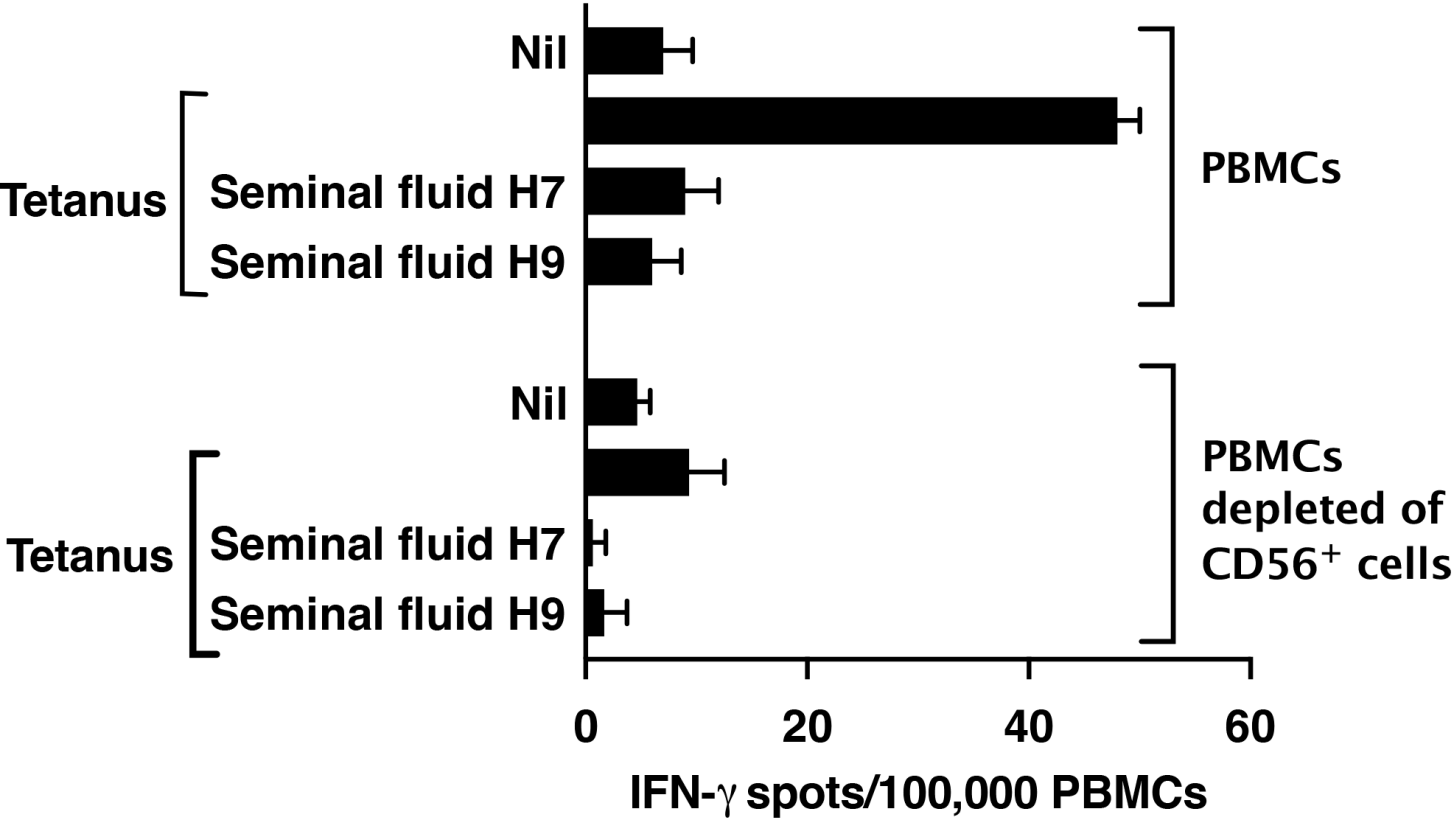
Depletion of NK cells from PBMCs markedly decreases IFN-γ expression in response to tetanus. NK cells in PBMCs were stained with FITC antibody to CD56 and depleted by flow sorting. Post-sort analysis demonstrated 100% depletion. PBMCs depleted or not of CD56^+^ cells were then analysed by IFN-γ ELISpot in response to tetanus (10Lfu/ml) or anti-CD3/28 Dynabeads as described in Methods.

Suppression by seminal fluid of the Jurkat CD4^+^ T-cell leukaemia line (see **Figure 3B**) can be attributed to its expression of Siglec-7 (**Supplementary Figure 5**). However, only a very small fraction of primary human T cells, specifically CD8^+^ T cells, are known to express Siglec-7 (29). We confirmed that seminal fluid had no direct effect on purified primary CD4^+^ T cells stimulated though the T-cell receptor by plate-bound anti-CD3/28 antibodies (**Figure 5**). An explanation is therefore needed for how Siglec-7-mediates suppression by seminal fluid of tetanus-induced IFN- γ production in PBMCs (see **Figure 4B**). Siglec-7 is expressed predominantly by antigen-presenting cells (APCs), namely monocytes and dendritic cells (DCs) (27), and by NK cells (29). NK cells have also been reported to act as APCs for T cells (30, 31). The usual sources of IFN-γ after antigen exposure are T cells activated by APCs and NK cells activated indirectly by APC-derived IL-12, IL-15 and IL-18 (reviewed in 32). Therefore, suppression of IFN-γ in PBMCs in response to tetanus could be an indirect effect on T cells or NK cells via APCs or a direct effect on NK cells. Consistent with a major contribution of NK cells to IFN-γ production, their depletion resulted in an 80% decrease in IFN-γ expression by PBMCs in response to tetanus, without changing the T-cell response to anti-CD3/28 (**Figure 6**).

**Figure 7:**
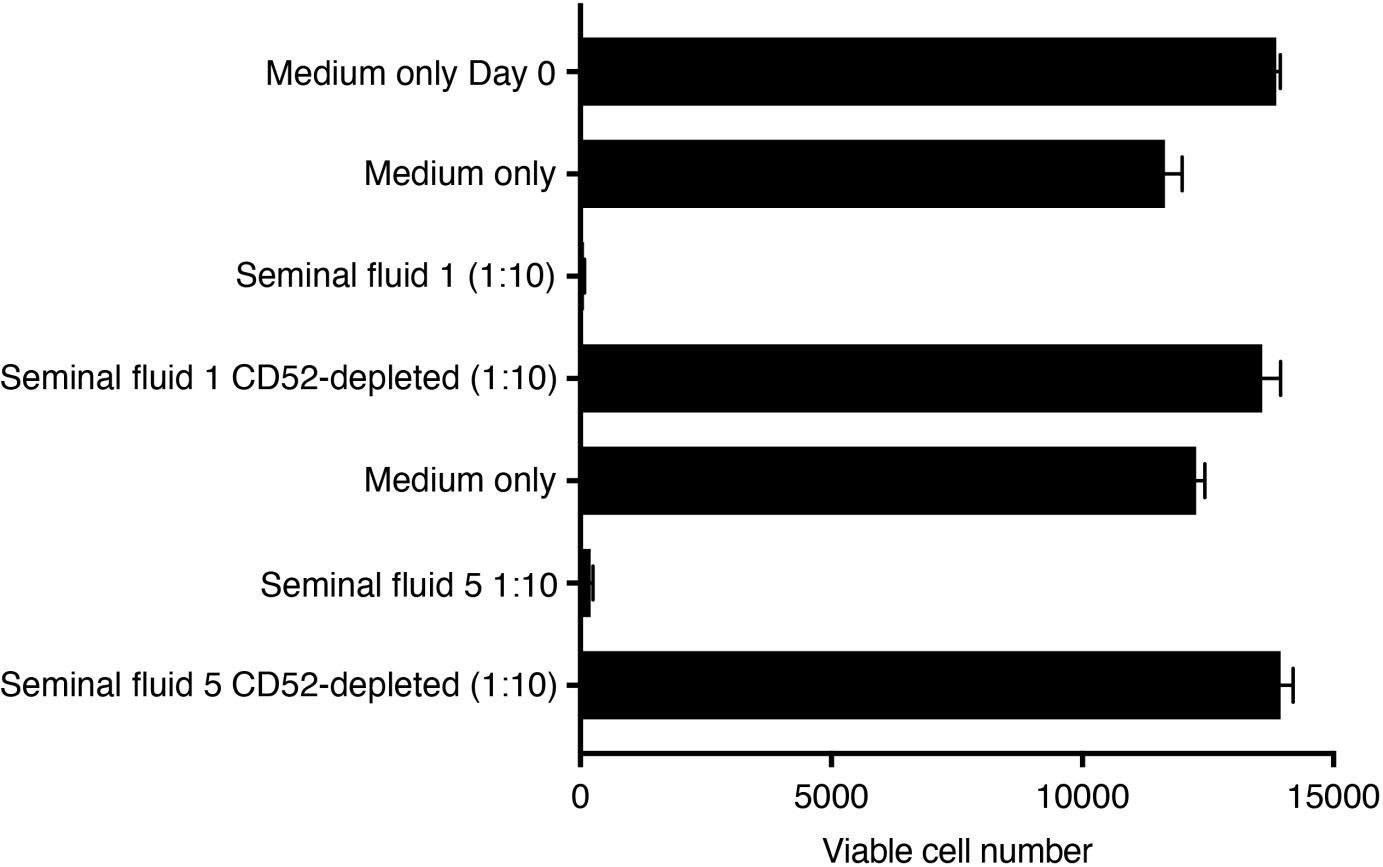
Seminal fluid CD52 inhibits expansion of primary human NK cells. Untouched NK cells were purified by negative immunomagnetic selection from freshly prepared PBMCs and suspended in NK MACS medium containing 5% human AB serum. Cells were seeded at 15,000 cells/well in round-bottom 96-well plates in a total volume of 100 μl in the presence of IL-15 (final 20ng/ml) and medium only, or seminal fluid at 1:10 dilution depleted or not depleted of CD52. After 96 h, viable cells were enumerated against cell counting beads in a flow cytometer.

NK cells are the predominant immune cell in the non-pregnant human endometrium and at the site of implantation of the embryonic placental trophoblast (33). To confirm that NK cells are a target of seminal fluid CD52, we incubated freshly isolated blood NK cells in IL-15 supplemented expansion medium in the presence of seminal fluid depleted or not of CD52 and quantified viable NK cells after 72-96 h. Seminal fluid markedly decreased NK cell proliferation, but seminal fluid depleted of CD52 had no effect (**Figure 7**).

## Discussion

We provide evidence that soluble CD52, acting via the immune suppressive receptor Siglec-7, accounts for T cell and NK cell suppression by seminal fluid. Suppression of T-cell responses to antigen (tetanus) by multiple different seminal fluid samples was not observed in the presence of an antibody (CF1D12) against the bioactive terminal glycan of CD52 (13) or after immunodepletion of CD52 from seminal fluid with alemtuzumab, a humanized monoclonal antibody against the C-terminus-GPI junction of CD52 (13). In addition, CD52 in seminal fluid, evidently in relative excess, was complexed with the danger-associated molecular pattern (DAMP) protein, HMGB1. Previously, we found that immune suppression by lymphoid CD52, acting via Siglec-10 on activated T cells, required its pre-complexing with HMGB1 (22). Sequestration of HMGB1 by CD52 may be a means to nullify the inflammatory effects of HMGB1 Box B (22). The requirement for HMGB1, which is present in serum (23), may explain an early observation (6) that suppression of T-cell activation by human seminal fluid required the presence of bovine serum in the medium. Lymphoid CD52 complexed to HMGB1 triggers the cytoplasmic immunoreceptor tyrosine-based inhibitory motif (ITIM) of Siglec-10 to recruit the Src homology 2 (SH2) domain-containing protein tyrosine phosphatase (SHP1), which dephosphorylates and inactivates the T cell receptor (20). The same mechanism has been described for Siglec-7 (34), which we show here interacts with seminal fluid CD52. Notably, of a range of Siglecs expressed on human sperm, Siglec-7 was conspicuously absent (35), which we suggest would allow sperm-expressed CD52 to avoid interaction in cis with Siglec-7 (36), thereby protecting sperm from inhibition by CD52 in seminal fluid.

CD52 was detected in a range of seminal fluid concentrations out to >1:1,000 dilution. This variation may reflect donor factors, but because samples were provided anonymously we were not able to relate CD52 concentration or function to clinical parameters. Nevertheless, it has been reported that CD52 expression is reduced in spermatozoa from sub-fertile men (37). Our samples were obtained from men attending an infertility clinic and therefore the extent to which they are representative of seminal fluid in general is a caveat. The CD52 sialoglycan on sperm has been shown to be the antigenic target of circulating anti-sperm immobilizing antibodies (19), detected in a minor proportion of infertile women (18). Many studies, dating back 70 years (38), have shown that anti-sperm antibodies are associated with decreased sperm concentration and function (39, 40). We did not measure anti-sperm antibodies or sperm concentration because our study was focused on the role of CD52 in mediating immune suppression by seminal fluid. It is possible that anti-sperm antibodies directed at CD52 in some samples may have affected the measurement of CD52 in seminal fluid, but this would not invalidate our finding that soluble CD52 mediates T and NK cell suppression by seminal fluid.

Given that CD52 appears to account for most of the suppressive effect of seminal fluid on T and NK cells, how is this reconciled with reports (3–9) of other suppressive factors in seminal fluid? Immune tolerance to sperm in the female is critically important for reproduction and it would not be surprising if several seminal fluid components were involved and acted in concert, such as those reported to condition the female response to sperm (3, 41, 42). However, with the possible exception of prostaglandins, there is little evidence that other seminal fluid components directly suppress T or NK cells. Suppression of lymphocyte and NK cell function by seminal fluid was attributed to PGEs (3–5, 7) but the evidence was inferential, not direct, and the relative contribution of PGEs was not clear. In addition, the original observation that lymphocyte suppression by seminal fluid was associated with prostasomes (10) has remained unexplained. Prostasomes are now known to be heterogenous EVs, and those shown to contain CD52 (11) are most likely derived from the epididymal epithelium (15, 42). Our findings provide an explanation for the prostasome effect, although we found that immune suppressive activity was mainly associated with the soluble fraction of seminal fluid. The original study (10) also found activity in the soluble fraction, but fractionated seminal fluid initially by ammonium sulphate precipitation, which we avoided. Our findings do not exclude the possibility that seminal fluid components other than CD52 may modulate immunity measured in different ways. For example, TGF-β1 and other members of the TGF-β family in seminal fluid have been shown to have profound regulatory and tolerogenic effects on dendritic cells, macrophages and lymphocytes, leading to the expansion of regulatory T cells (Treg) in the female (9, 42). Similarly, prostaglandin E (PGE) and 19-OH-PGE 1 and 2 in seminal fluid may contribute, in concert with TGF-β, to the induction of Treg and may have other anti-inflammatory actions (42). The obvious limitation of this human study is that it was performed *in vitro*, and could not elucidate the role or contribution of seminal fluid CD52 to immune tolerance to sperm *in vivo*. To address this question, investigation in animal models, beyond the scope of the current study, will be required. Nevertheless, we conclude that CD52 in seminal fluid mediates T cell and NK cell suppression by interacting with the inhibitory receptor, Siglec-7, on APCs and NK cells. This finding adds an important new dimension to our understanding of the critical role of seminal fluid in facilitating reproduction.

## Data Availability

The original contributions presented in the study are included in the article/**Supplementary Material**. Further inquiries can be directed to the corresponding author.
