# Supplementary figures and images for: Soluble CD52 mediates immune suppression by human seminal fluid
Source: Front Immunol. 2024 Dec 16;15:1497889. doi: 10.3389/fimmu.2024.1497889 (PMC11682959; doi:10.3389/fimmu.2024.1497889)

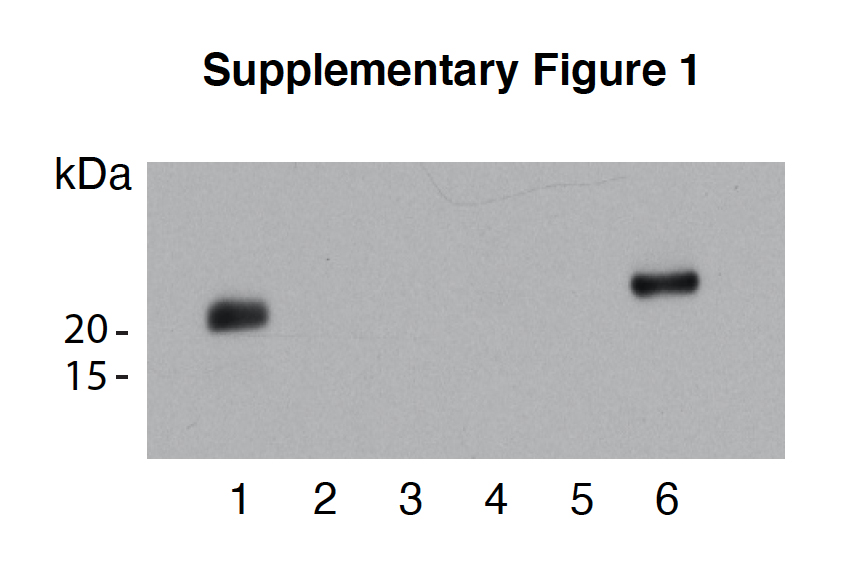

Supplement: Supplementary file 1 [file Image1.jpeg]

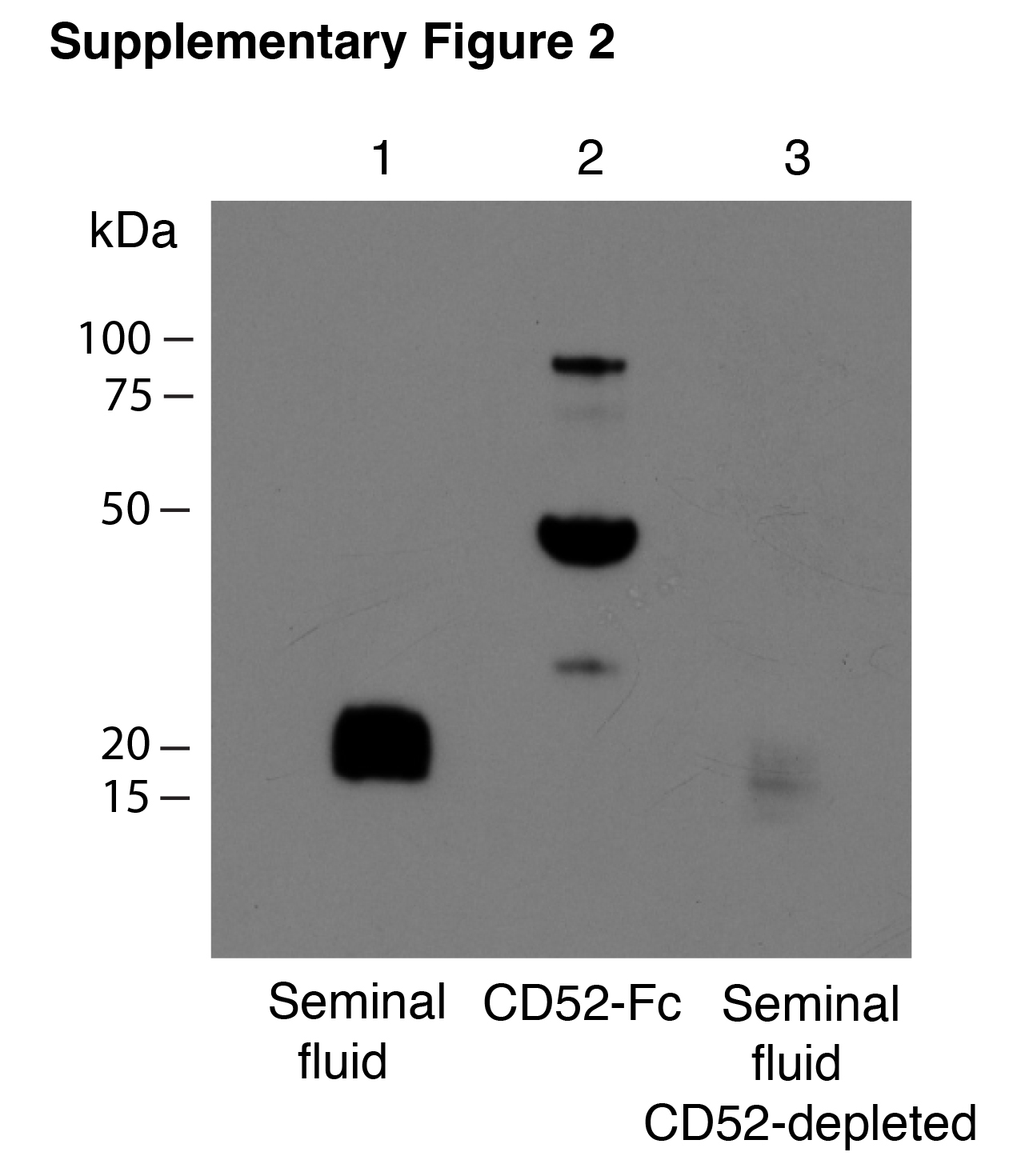

Supplement: Supplementary file 2 [file Image2.jpeg]

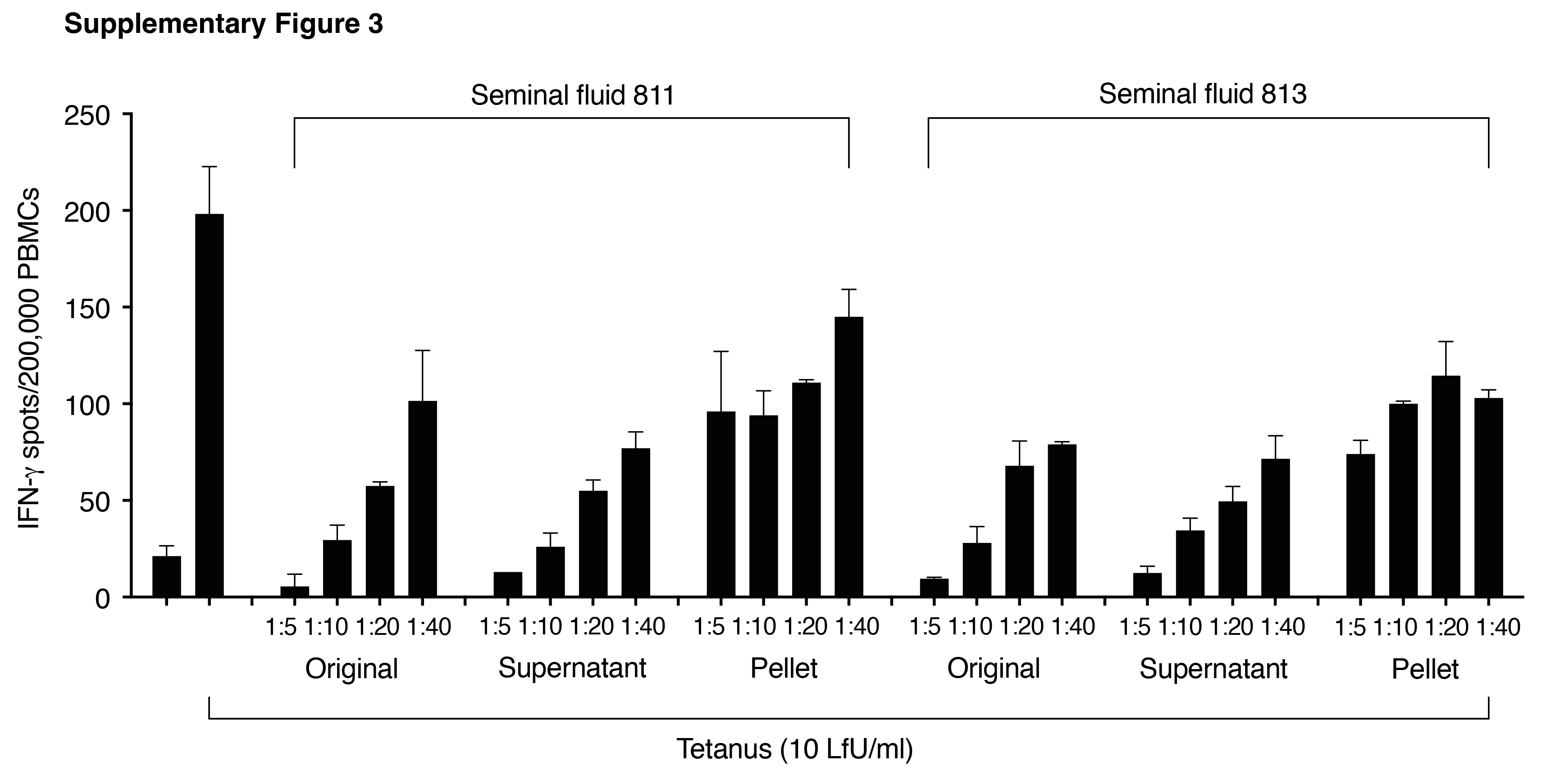

Supplement: Supplementary file 3 [file Image3.jpeg]

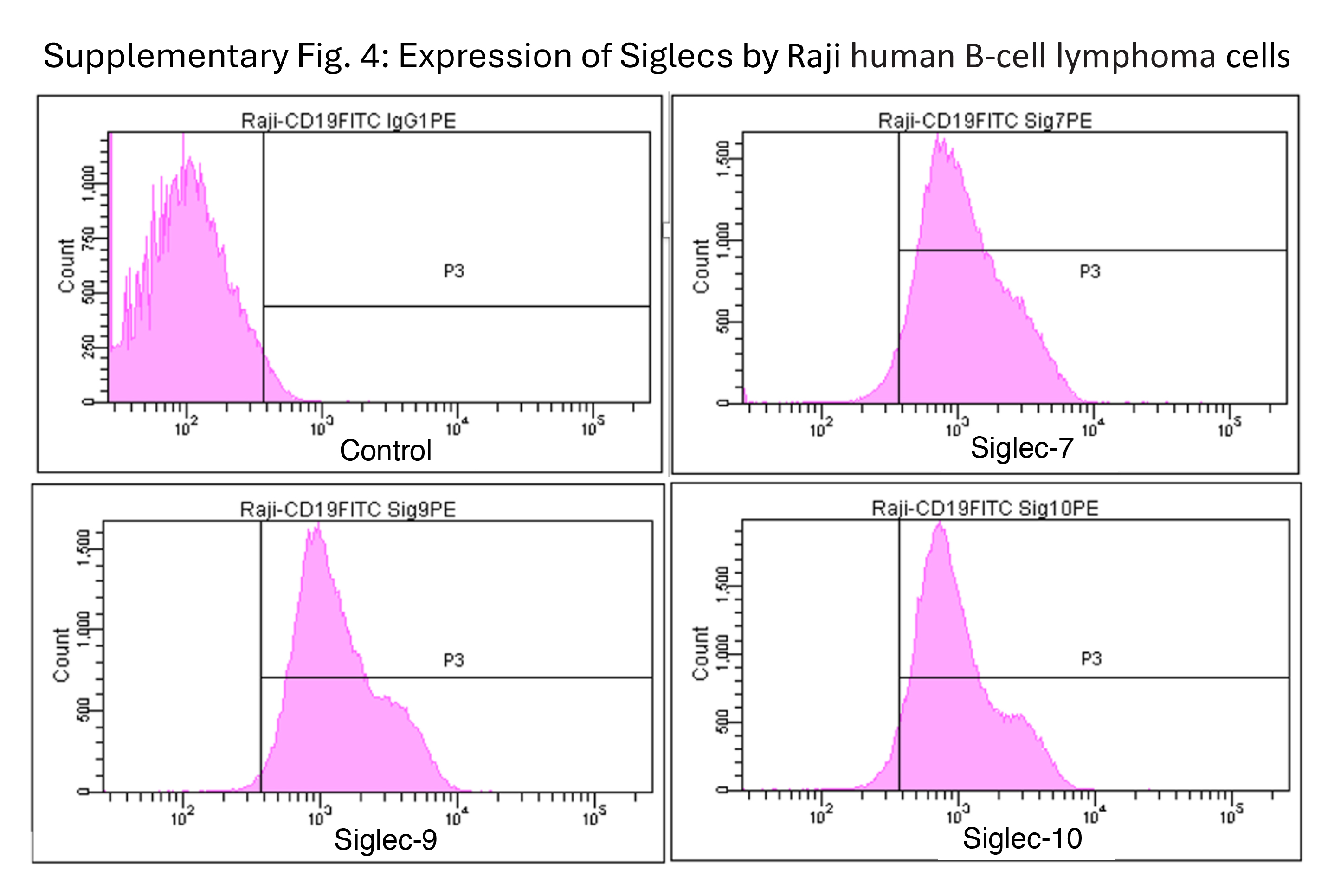

Supplement: Supplementary file 4 [file Image4.jpeg]
